# Quantitative comparisons of ultra-widefield images of model eye obtained with Optos^®^ 200Tx and Optos^®^ California

**DOI:** 10.1186/s12886-019-1125-y

**Published:** 2019-05-17

**Authors:** Yu Kato, Makoto Inoue, Akito Hirakata

**Affiliations:** Kyorin Eye Center, Kyorin University School of Medicine, 6-20-2 Shinkawa, Mitaka, Tokyo 181-8611 Japan

**Keywords:** Wide-field image, Angle, Contrast, Scanning laser ophthalmoscope

## Abstract

**Background:**

To compare the quality of the ultra-widefield images acquired by Optos® 200Tx to those acquired by Optos® California.

**Methods:**

Images of the posterior surface of a Gullstrand’s model eye obtained by Optos® 200Tx were compared to those obtained by Optos® California in terms of the angular field of view and the symmetry of the image, i.e., vertical and horizontal aspect ratios at the center (0°) and at the periphery (40° and 80°) in each direction. In addition, we compared the enlargement of the image on the posterior surface as the square ratio, and the differences in the contrasts.

**Results:**

No significant differences were detected in the angular field of view between the two instruments. The aspect ratios showed that the Optos® California had more symmetrical images than the Optos® 200Tx at the center (0.98 vs 0.89, *P* < 0.01) and at the 40° periphery (0.93–1.04, △0.11 vs 0.79–1.01, △0.22) and 80° periphery (0.81–1.25, △0.44 vs 0.42–1.12, △0.70) in each direction. The amplitude of the square ratio of the Optos® California was smaller at 40° periphery (1.16–1.28, △0.12 vs 1.06–1.37, △0.31) and 80° periphery (2.12–2.46, △0.34 vs 1.14–3.29, △2.15). The contrast of the Optos® California images was significantly higher in the posterior pole (0.09 vs 0.12, *P* < 0.01), upper (0.07 vs 0.03, *P* < 0.01), and right (0.12 vs 0.07, *P* < 0.01) peripheries.

**Conclusion:**

Optos® California can record equal angular widefield images to Optos® 200Tx and more symmetrical images with higher contrast in the posterior pole, upper and right peripheries.

## Background

Ultra-widefield imaging of the ocular fundus allows clinicians to evaluate the retina and choroid far beyond the equator in a single image [1–3]. The Optos® (Optos, Marlborough, MA, USA) is an ultra-widefield imaging system using a scanning laser ophthalmoscope. The widefield images are obtained with two laser light sources of wavelengths 532 nm (green) and 633 or 635 nm (red) [4]. The images obtained by the two wavelengths can be viewed separately or combined to yield a semi-realistic color image. The design of the ellipsoid mirror of the Optos® makes it possible to obtain ultra-widefield images of approximate 200 degrees horizontally without pupillary dilatation.

Recently, an ultra-widefield scanning laser ophthalmoscope, Optos® California, has become commercially available. The Optos® California includes a ProView™ software (https://www.optos.com/en/products/our-software-products/) which can obtain widefield images in a consistent geometry that accurately represents the anatomical features of the retina. New proprietary optical hardware is embedded in the Optos® California that optimizes its resolution throughout the retinal regions which results in better clarity of the periphery.

The purpose of this study was to compare the images of the posterior surface of a human model eye obtained by Optos® California to those obtained by Optos® 200Tx without the ProView™ software.

## Methods

**Fig. 1:**
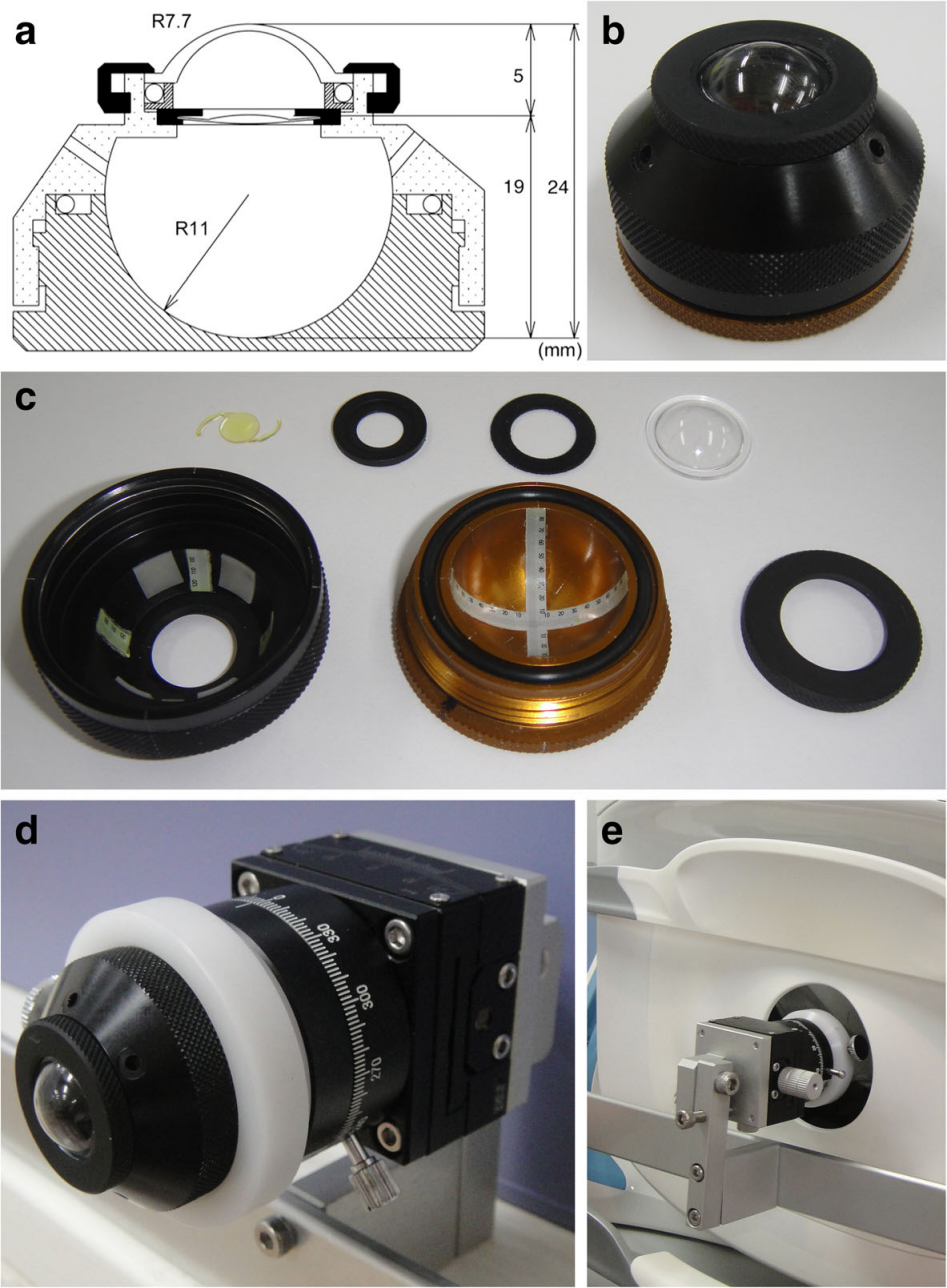
Photographs of the human model eye and schematic drawing of mode. **a. b.** The appearance of the model eye used to quantify the images with the Optos® 200Tx and Optos® California widefield scanning ophthalmoscopes. The model eye has an axial length of 24 mm. The body of the model eye is made of metal, and the cornea is made of polymethylmethacrylate. **c.** Angular scales are attached to the inner posterior surface of the model eye and the images of the angular scales are quantified. **d. e.** A jig is attached to fix the model eye in the appropriate position

**Fig. 2:**
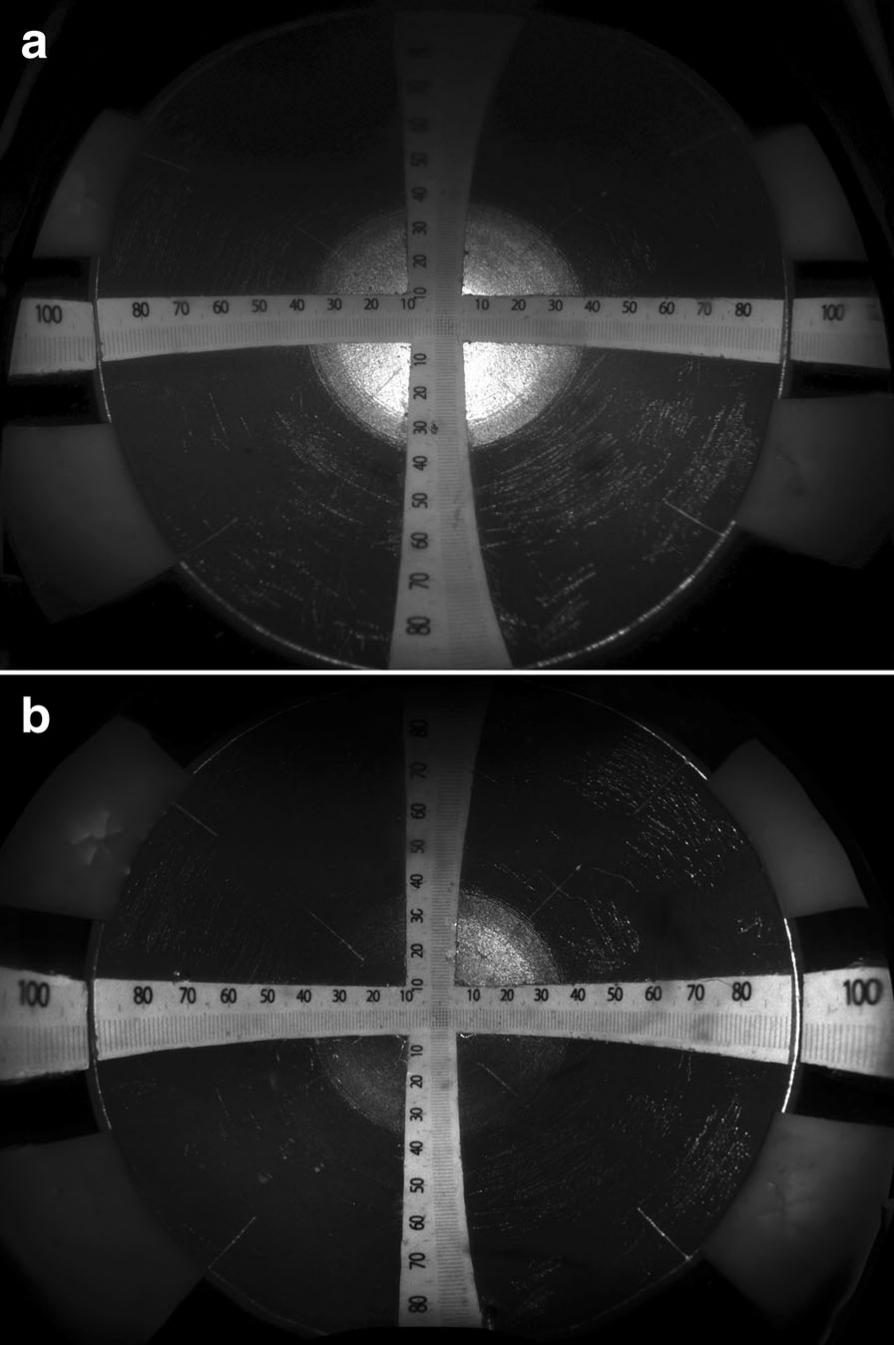
Fundus images obtained by the widefield scanning ophthalmoscope. Optos® 200Tx (**a**) and Optos® California (**b**). The length of the scale bar and interval of scale bars are measured to quantify the magnification in the peripheral part of the image

The quality of the images of the posterior surface of a model eye constructed based on the Gullstrand’s model of the human eye was determined (Fig. 1). The axial length of the model eye was 24.0 mm, the cornea was made of polymethylmethacrylate with the radius of curvature of the anterior surface of 7.70 mm and posterior surface of 7.46 mm. The diameter of the pupil of the model eye was 8.11 mm. An intraocular lens AcrySof® IQ (SN60WF, Alcon Laboratories, Inc., Fort Worth, TX, USA) of + 20.0 diopter was placed intracamerally. Two tapes marked in angles for the optics of the model eye was pasted on the posterior surface of the model eye in the vertical and horizontal positions. These were used to evaluate the angular field of view, the symmetry, and contrast of the images projected onto the posterior surface of the model eye. The model eye was filled with distilled water at room temperature. The model eye was mounted on the optical axis of the system with a jig and placed in the front of the ultra-widefield scanning laser ophthalmoscope (Fig. 1). The position of the model eye was adjusted to set the center of the posterior surface at the center of the image. The images of the posterior surface of the model eye were recorded with the Optos® 200Tx and the Optos® California 10 times, and the averaged values were used for the statistical analyses (Fig. 2). The device turns on a green light when the image of the posterior surface of the model eye is aligned properly. The model eye was placed in a position where the green light remained on throughout the experiment.

The images were converted to JPEG files of 3900 × 3072 pixels for the Optos® 200Tx or 4000 × 4000 pixels for the Optos® California. The images were analyzed with the Photoshop® CS5 image-editing software (Adobe Systems, San Jose, CA, USA). The exported images of the red-laser channel were analyzed because it had better contrast than both the green channel and the combined channels.

To measure the maximal angular field of view, the most peripheral readable marker on the vertical and horizontal scales was determined for each image. The directions of right and left were defined in terms of the direction of the model eye. The number of pixels of 5° scales of the center position of the posterior pole of 0° and at periphery of 40° and 80° from the center in each direction were also measured to quantify the aspect ratios and square ratios of the periphery. The aspect ratio was measured to evaluate the symmetry of the images as the horizontal and vertical aspect ratios. The number of pixels of 5° scales of the height at position of z° was set as the value of *h*_*z*_ (pixels), and that of width was set the value of *w*_*z*_ (pixels). The aspect ratio in the z° position was calculated as *h*_*z*_/*w*_*z*_. The square ratio was used to evaluate the enlargement of the image relative to the center of the posterior surface. A square size of 5° × 5° at the position of z° was set as the value of *S*_*z*_ and calculated as *h*_*z*_ x *w*_*z*_ (pixels^2^). The square ratio relative to the center of the posterior pole of the position of z° was calculated as *S*_*z*_/*S*_*0*_. Each value was measured by one of the authors (YK) who was masked to any identifiable information of each image.

The contrast of the images was graded on a 256 Gy steps scale at the center (0°) and at the periphery (80°) in each direction. The mean intensity in the central four pixels of the black stripe in the z° position was set as the value of *B*_*z*_, and that of the white stripe was set as the value of *W*_*z*_. The contrast in the position of z° was calculated as,

$$ \left({B}_z-{W}_z\right)/\left({B}_z+{W}_z\right) $$

### Statistical analyses

Statistical analyses were performed with the SPSS software (Chicago, III., USA). The significance of the differences among groups was determined by the Mann-Whitney U test. A *P* of < 0.05 was taken to be statistically significant.

## Results

**Fig. 3:**
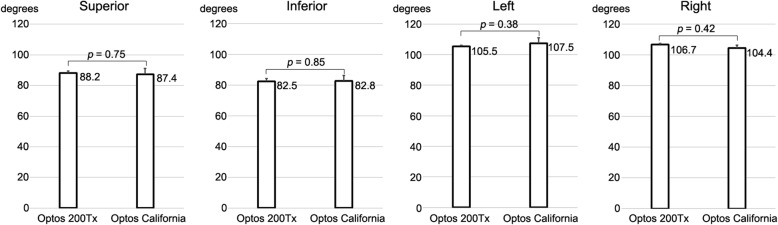
The maximal angular field of view of the Optos® 200Tx and Optos® California. No significant difference is detected for each direction

The maximal angular field of view of the images of the posterior surface of the model eye obtained with the Optos® 200Tx was 88.2 ± 1.1^°^ in the superior, 105.5 ± 0.5^°^ in the left, 82.5 ± 1.8^°^ in the inferior, and 106.7 ± 0.9^°^ in the right directions. Similarly, the maximal angular field of view of the images of the posterior surface by the Optos® California was 87.4 ± 3.6^°^ in the superior, 107.5 ± 3.4^°^ in the left, 82.8 ± 3.5^°^ in the inferior, and 104.4 ± 2.0^°^ in the right directions (Fig. 3). The differences in the maximal angular field of view for the two devices were not significant.

**Fig. 4:**
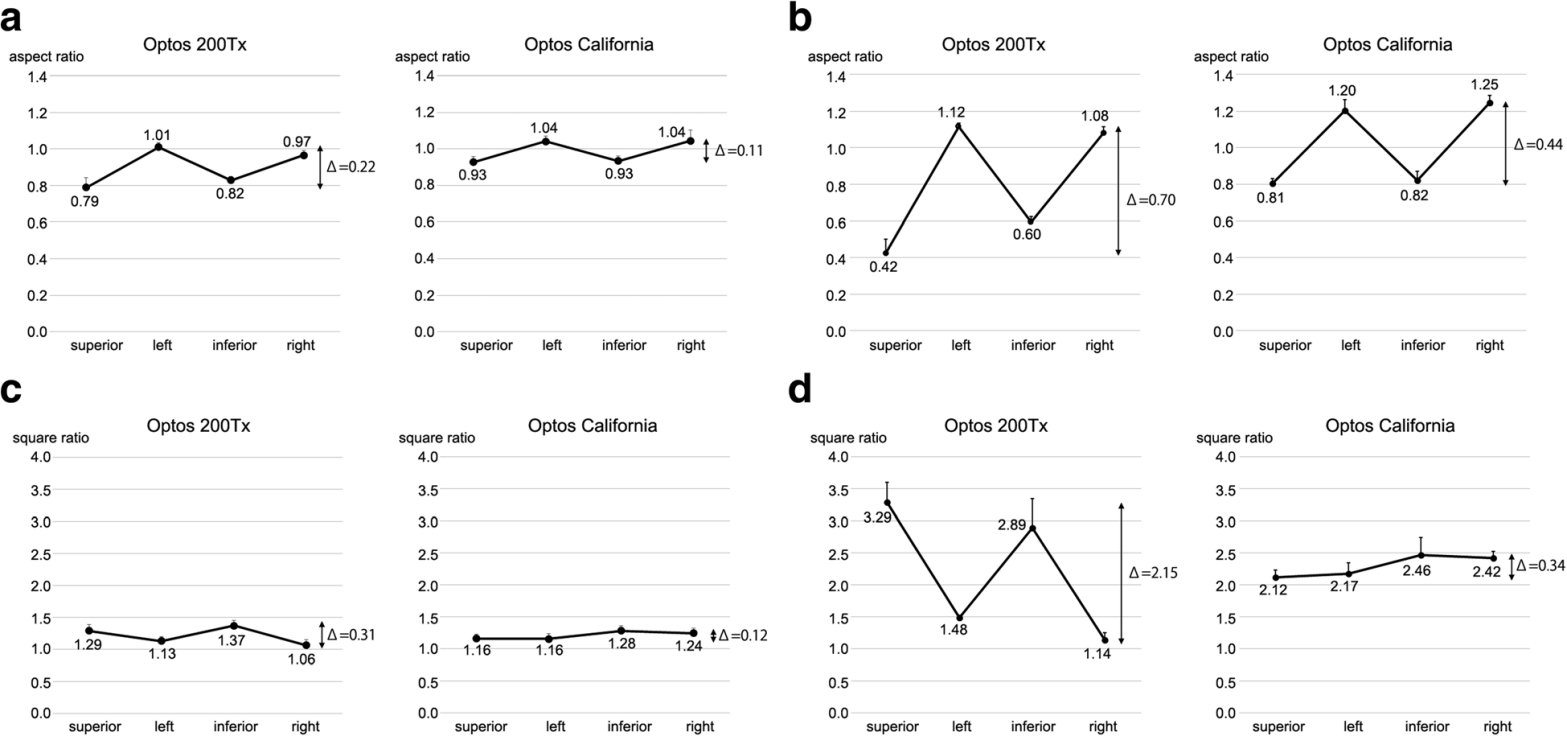
The aspect ratio and the square ratio at the periphery 40° and 80° obtained with Optos® 200Tx and Optos® California.**a.** The aspect ratio at the periphery 40° of Optos® California are settled in smaller amplitude of values than that of Optos® 200Tx (0.11 vs 0.22). **b.** The aspect ratio at the periphery 80° of Optos® California are settled in smaller amplitude of values than that of Optos® 200Tx (0.44 vs 0.70). **c.** The square ratio of Optos® California at the periphery 40° relative to the center of the posterior pole are settled in smaller amplitude of values than that of Optos® 200Tx (0.12 vs 0.31). **d.** The square ratio of Optos® California at the periphery 80° relative to the center of the posterior pole are settled in smaller amplitude of values than that of Optos® 200Tx (0.34 vs 2.15)

The aspect ratio of the images at the center of the posterior surface was 0.89 ± 0.01 for the Optos® 200Tx and 0.98 ± 0.02 for the Optos® California (*P* < 0.01). At 40° periphery, the aspect ratio of the Optos® 200Tx was 0.79 ± 0.05 in the superior, 1.01 ± 0.02 in the left, 0.82 ± 0.02 in the inferior, and 0.97 ± 0.02 in the right directions. The aspect ratio of Optos® California at 40° periphery was 0.93 ± 0.03 in the superior, 1.04 ± 0.03 in the left, 0.93 ± 0.03 in the inferior, and 1.04 ± 0.06 in the right directions. The amplitudes of the Optos® California were significantly smaller than those of Optos® 200Tx (△0.22 ± 0.05 vs △0.11 ± 0.05, *P* < 0.01, Fig. 4a). At 80° periphery, the aspect ratio of the Optos® 200Tx was 0.42 ± 0.08 in the superior, 1.12 ± 0.02 in the left, 0.60 ± 0.03 in the inferior, and 1.08 ± 0.03 in the right directions. The aspect ratio of Optos® California at 80° periphery was 0.81 ± 0.03 in the superior, 1.20 ± 0.06 in the left, 0.82 ± 0.05 in the inferior, and 1.25 ± 0.04 in the right directions. The amplitudes of the Optos® California were significantly smaller than that of Optos® 200Tx (△0.70 ± 0.07 vs △0.44 ± 0.06, *P* < 0.01, Fig. 4b).

At 40° periphery, the square ratio relative to the center of the posterior surface of the Optos® 200Tx images was 1.29 ± 0.10 in the superior, 1.13 ± 0.07 in the left, 1.37 ± 0.09 in the inferior, and 1.06 ± 0.09 in the right directions. The square ratio at 40° periphery relative to the center of the posterior surface of the images of the Optos® California was 1.16 ± 0.07 in the superior, 1.16 ± 0.08 in the left, 1.28 ± 0.08 in the inferior, and 1.24 ± 0.08 in right directions. The amplitudes of the square ratios of the Optos® California were significantly smaller than that of the Optos® 200Tx (△0.31 ± 0.11 vs △0.12 ± 0.06, *P* < 0.01, Fig. 4c).

At 80° periphery, the square ratio relative to the center of the posterior surface of the Optos® 200Tx images was 3.29 ± 0.31 in the superior, 1.48 ± 0.05 in the left, 2.89 ± 0.46 in the inferior, and 1.14 ± 0.11 in the right directions. The square ratio at 80° periphery relative to the center of the posterior surface of the images of the Optos® California was 2.12 ± 0.11 in the superior, 2.17 ± 0.17 in the left, 2.46 ± 0.28 in the inferior, and 2.42 ± 0.11 in right directions. The ratios of the Optos® California were significantly smaller than that of the Optos® 200Tx (△2.15 ± 0.42 vs △0.34 ± 0.21, *P* < 0.01, Fig. 4d).

**Fig. 5:**
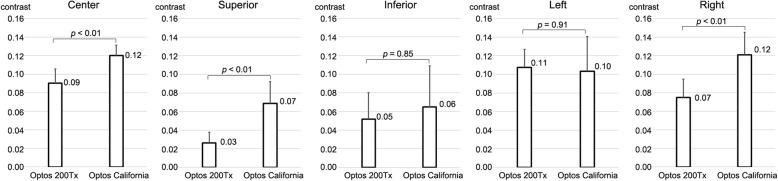
The contrast of the images at the center and periphery 80° obtained with Optos® 200Tx and California. The contrast of the images obtained with Optos® California is significantly better at the center (0.12 vs 0.09), superior (0.07 vs 0.03) and right (0.12 vs 0.07) direction

The contrast of the images at the center of the posterior surface was 0.09 ± 0.02 for the Optos® 200Tx and 0.12 ± 0.01 for the Optos® California (*P* < 0.01). At 80° periphery, the contrast of the images obtained with Optos® California was significantly better in the superior at 0.03 ± 0.01 than the 0.07 ± 0.02 obtained with the Optos® 200Tx (*P* < 0.01). This also held for the images in the right direction (0.07 ± 0.02 vs 0.12 ± 0.02, *P* < 0.01, Fig. 5).

## Discussion

The newly developed ProView™ software installed in the Optos® California instrument can construct widefield images with in a consistent geometry that accurately represent the anatomical features of the retina. At the commercial site of Optos® Incorporated (https://www.optos.com), ProView™ is introduced as the software which normalizes the inherent bias which occurs when curved surfaces, such as the retina, are displayed on a flat plane. Our results showed that the Optos® California covered approximately the same angular extent of the ocular fundus as the Optos® 200Tx without the ProView™ software. In addition, the aspect ratio of the Optos® California at the center was closer to 1.0, and the ratio at the periphery was smaller in amplitude than that of Optos® 200Tx with horizontally elongated images. These characteristics indicate that the distortions and the deformities of the central and the peripheral images of Optos® California are less than that of Optos® 200Tx. Similarly, the square ratio relative to the center of posterior pole of Optos® California at the periphery had a smaller amplitude than that of Optos® 200Tx. This also indicates that the distortions of the images of Optos® California at the periphery is less than that of Optos® 200Tx. Oishi and associates reported that the images at the periphery of the Optos® 200Tx are distorted to a similar degree [5] probably because the device uses an ellipsoid mirror rather than a spherical one. The ProView™ software adjusts for the distortion of the images caused by the ellipsoid mirror at the periphery by image processing calculations.

The contrast of the Optos® California images was better than that of Optos® 200Tx in the center and also in the superior and right directions. Oishi and associates also reported that the contrasts of the Optos® 200Tx images were the best in the left region and worse in the superior and right regions [5]. Consequently, the contrast of the images in the superior and right directions of the periphery were better in the Optos® California which might be due to the new proprietary hardware.

The evaluation of the peripheral retina is important for screening [6] and for evaluating diseases including diabetic retinopathy [7], retinal vein occlusion [8], age-related macular degeneration [9, 10], retinopathy of prematurity [11], uveitis [12–14], and retinal/choroidal dystrophy [15, 16]. By examining less distorted images of the periphery, the accuracy of the diagnosis and the identifications of alterations will likely improve. In addition, evaluations of the peripheral non-perfused capillary areas should consider the effect of enlargements represented by the square ratio [17, 18].

This study has several limitations. The size of the model eye is fixed so that variations such as that in hyperopic or myopic eyes cannot be investigated. Similarly, the conditions of phakic and aphakic eyes were also not investigated and further analysis is required.

In conclusion, Optos® California can record widefield images comparable to those obtained by the Optos® 200Tx, but are more symmetrical with higher contrast in the posterior pole, upper and right peripheries. The distortion of the images caused by the ellipsoid mirror images can be reduced by the processing calculations. However, enlarged images of the periphery relative to the center due to widefield imaging still need to be considered to evaluate the entire retinal fundus.

## Abbreviations

JPEG: Joint Photographic Experts Group
SPSS: Statistical Package for Social Science

## References

[1] Friberg TR, Pandya A, Eller AW (2003). Non-mydriatic panoramic fundus imaging using a non-contact scanning laser-based system. Ophthalmic Surg Lasers Imaging.

[2] Anderson L, Friberg TR, Singh J (2007). Ultrawide-angle retinal imaging and retinal detachment. Semin Ophthalmol.

[3] Mackenzie PJ, Russell M, Ma PE, Isbister CM, Maberley DA (2007). Sensitivity and specificity of the optos optomap for detecting peripheral retinal lesions. Retina.

[4] Kernt M, Schaller UC, Stumpf C, Ulbig MW, Kampik A, Neubauer AS (2010). Choroidal pigmented lesions imaged by ultra-wide-field scanning laser ophthalmoscopy with two laser wavelengths (Optomap). Clin Ophthalmol.

[5] Oishi A, Hidaka J, Yoshimura N (2014). Quantification of the image obtained with a wide-field scanning ophthalmoscope. Invest Ophthalmol Vis Sci.

[6] Heussen FM, Tan CS, Sadda SR (2012). Prevalence of peripheral abnormalities on ultra-widefield greenlight (532 nm) autofluorescence imaging at a tertiary care center. Invest Ophthalmol Vis Sci.

[7] Soliman AZ, Silva PS, Aiello LP, Sun JK (2012). Ultra-wide field retinal imaging in detection, classification, and management of diabetic retinopathy. Semin Ophthalmol.

[8] Spaide RF (2013). Prospective study of peripheral panretinal photocoagulation of areas of nonperfusion in central retinal vein occlusion. Retina.

[9] Reznicek L, Wasfy T, Stumpf C, Kampik A, Ulbig M, Neubauer AS, Kernt M (2012). Peripheral fundus autofluorescence is increased in age-related macular degeneration. Invest Ophthalmol Vis Sci.

[10] Tan CS, Heussen F, Sadda SR (2013). Peripheral autofluorescence and clinical findings in neovascular and non-neovascular age-related macular degeneration. Ophthalmology.

[11] Patel CK, Fung TH, Muqit MM, Mordant DJ, Brett J, Smith L, Adams E (2013). Non-contact ultra-widefield imaging of retinopathy of prematurity using the Optos dual wavelength scanning laser ophthalmoscope. Eye.

[12] Leder HA, Campbell JP, Sepah YJ, Gan T, Dunn JP, Hatef E, Cho B, Ibrahim M, Bittencourt M, Channa R, Do DV, Nguyen QD (2013). Ultra-wide-field retinal imaging in the management of non-infectious retinal vasculitis. J Ophthalmic Inflamm Infect.

[13] Campbell JP, Leder HA, Sepah YJ, Gan T, Dunn JP, Hatef E, Cho B, Ibrahim M, Bittencourt M, Channa R, Do DV, Nguyen QD (2012). Wide-field retinal imaging in the management of noninfectious posterior uveitis. Am J Ophthalmol.

[14] Reznicek L, Seidensticker F, Stumpf C, Kampik A, Thurau S, Kernt M, Neubauer A (2014). Systematic analysis of wide-field fundus autofluorescence (FAF) imaging in posterior uveitis. Curr Eye Res.

[15] Yuan A, Kaines A, Jain A, Reddy S, Schwartz SD, Sarraf D (2010). Ultra-wide-field and autofluorescence imaging of choroidal dystrophies. Ophthalmic Surg Lasers Imaging.

[16] Oishi A, Ogino K, Makiyama Y, Nakagawa S, Kurimoto M, Yoshimura N (2013). Wide-field fundus autofluorescence imaging of retinitis pigmentosa. Ophthalmology.

[17] Spaide RF (2011). Peripheral areas of nonperfusion in treated central retinal vein occlusion as imaged by wide-field fluorescein angiography. Retina.

[18] Sawada O, Ichiyama Y, Obata S, Ito Y, Kakinoki M, Sawada T, Saishin Y, Ohji M (2018). Comparison between wide-angle OCT angiography and ultra-wide field fluorescein angiography for detecting non-perfusion areas and retinal neovascularization in eyes with diabetic retinopathy. Graefes Arch Clin Exp Ophthalmol.

